# Alveolar socket healing in 5-lipoxygenase knockout aged female mice treated or not with high dose of zoledronic acid

**DOI:** 10.1038/s41598-021-98713-2

**Published:** 2021-10-01

**Authors:** Ramez H. Mahmoud, Claudia C. Biguetti, Gustavo B. Simionato, Isabela C. Custódio, Raquel B. P. Silva, Marco A. H. Duarte, Leonardo P. Faverani, Edilson Ervolino, Walid D. Fakhouri, Mariza A. Matsumoto

**Affiliations:** 1 Department of Basic Sciences, School of Dentistry, São Paulo State University (UNESP), Araçatuba, São Paulo Brazil; 2 Department of Bioengineering, University of Texas at Dallas, Richardson, TX USA; 3 Department of Health Sciences, Centro Universitário Sagrado Coração, Bauru, São Paulo Brazil; 4 Bauru School of Dentistry, University of São Paulo, FOB-USP, Bauru, São Paulo Brazil; 5 Department of Surgery, School of Dentistry, São Paulo State University (UNESP), Araçatuba, São Paulo Brazil; 6 Center for Craniofacial Research, University of Texas Health Science Center at Houston, School of Dentistry, Houston, TX USA

**Keywords:** Cells, Endocrine system, Oral anatomy, Preclinical research, Anatomy, Medical research, Risk factors

## Abstract

This study investigated the role 5-lypoxigenase (5-LO) on alveolar socket healing in aged female mice treated with zoledronic acid (ZL). Forty 129/Sv female mice (64–68 weeks old), 20 wild type (WT) and 20 5-LO knockout (5LOKO) were equally distributed according to ZL treatment: WT Control, WT ZL, 5LOKO Control, and 5LOKO ZL. ZL groups were treated with an intraperitoneal injection of 250 µg/Kg of ZL, while controls were treated with saline. Treatments were administered once a week, starting four weeks before surgery for tooth extraction and until 7 and 21 days post-surgery. Mice were euthanized for a comprehensive microscopic analysis (microCT, histomorphometry and immunohistochemistry). WT ZL mice presented intense inflammatory infiltrate (7 days), delayed bone formation (21 days), reduced collagenous matrix quality, and a deficiency in Runx-2 + , TRAP + , and macrophages as compared to controls. 5LOKO ZL animals presented decreased number of Runx-2 + cells in comparison to 5LOKO Control at 7 days, but no major changes in bone healing as compared to WT or 5LOKO mice at 21 days. The knockout of 5LO favored intramembranous bone healing in aged female mice, with a direct impact on inflammatory response and bone metabolism on the development of ONJ-like lesions.

## Introduction

The effects of aging in female leads to a prominent reduction of sex hormones^1^. Around fifth decade of life, women experience menopause as a remarkable transition phase that begins with the end of the menstrual cycle and lasts for several decades. Menopause predisposes women to multiple psychogenic/behavioral events and increases the risks of osteoporosis. During women’s reproductive period, estrogens promote osteoblast development instead of adipocytes, and increase their proliferative capacity and the synthesis of proteins, such as osteoprotegerin (OPG). Also, estrogens induce osteoclasts apoptosis and decrease the production of RANKL. The drastic reduction of estrogens, especially E2, that begins in perimenopause, results in an important deficit of bone mass and mineral content, as well consequent increased fracture risk and deficient healing capacity^2^. This condition continues during and after menopause. In order to minimize the effects of hormone drastic decrease on bone metabolism, some therapeutic options are available as calcium and hormone reposition, the use of selective estrogen receptor modulators (SERMs), and antiresorptives nitrogen-containing bisphosphonates (nBPs). Furthermore, these antiresorptive drugs and new alternatives have been also used or tested for patients under risk to develop severe osteoporosis due to continuous use of corticosteroid medication^3^. Among these drugs, zoledronic acid is a high potent nBP used as the first choice for the treatment of severe osteoporosis, improving bone density and preventing vertebra fracture and collapse in elderly women^4,5^. Zoledronic acid has been prescribed for all conditions above cited, and also to treat bone damage associated with oncologic diseases, such as metastatic breast cancer or multiple myeloma^6^. However, these drugs are directly related with the development of medication related osteonecrosis of the jaws (ONJs), especially when patients are exposed to invasive oral procedures such as tooth extractions and nBPs administrated by parenteral route^7,8^. Thus, given the increasing demand of elderly population in need of surgical procedures for oral rehabilitation^9^, the understanding of the detrimental role of zoledronic acid on alveolar bone healing is critical for ONJ prevention and treatment.

ONJ onset has been associated to the action of nBPs on bone cells, inducing osteoclasts precursors and active osteoclasts into apoptosis, and consequently reducing bone remodeling on maxillary bones^10,11^. Furthermore, nBPs also affect osteocytes, blocking apoptosis of the damaged cells, and leading them to cell senescence^12,13^. Consequently, these bone cells have a disruption in their function and capacity of reacting to stimulus that damage bone matrix^10^. ONJ pathogeny has been linked to reduced capacity of bone cells to repair a damaged bone matrix. These changes in bone turnover also result in modifications on jaw’s bones microarchitecture, such as increased trabecular density of the alveolar bone and increased density of cortical bone after long term of nBP administration^14^. Additionally, there was some evidence that polymorphisms in specific genes related to bone resorption (i.e. farnesyl pyrophosphate synthase gene) may increase the risk of developing ONJ^14^. However, some investigations shed light on effects of nBPs disturbing cells from specific sites of skeleton^15–17^ as well immune/inflammatory response^18,19^. A disrupted inflammatory response caused by nBPs clearly play a significant role in the development of ONJ onset, with altered production of proinflammatory cytokines and impairment of the immune response to secondary infection^14^.

Osteoclasts and macrophages are both derived from monocytic lineage, and consequently, they share a number of signaling and regulating molecules^20^. Like the osteoclasts, macrophages are also able to internalize nBPs particles^11,12^. It is important to consider that macrophages play important role in both bone physiology and repair, considering the resident macrophages (osteomacs)^21^, and those that are recruited into bone healing sites^21–23^. A study using a model of multiple myeloma in mice have shown that treatment with ZL can cause a disturbance in macrophages polarization and inflammatory response^19^. This same previous study demonstrated an increased expression of IL17 and abundance of macrophages in the oral mucosa bordering the non-healing extraction sockets of patients treated with IV nBPs^19^. However, how inflammatory cells and its mediators contribute to ONJ onset is still poorly understood, as well other associated risk factors such as age and gender^24,25^.

Among several risk factors for ONJs onset and development, the risk appears to increase in the presence of the following factors: IV nBP (dose and duration dependent), tooth extraction, and older age^14,26,27^. These findings reinforce the need of understanding how inflammatory mediators regulate bone healing in this susceptible population. Our group has characterized a model of ONJ-like lesions post tooth extraction in aged female 129 Sv mice (17 months) continuously treated with ZL by parenteral administration^25^, since ZL is a highly recommended drug for severe postmenopausal osteoporosis and/or bone damage related to breast cancer treatment in these population^28^. Animals treated with ZL developed typical ONJ-like lesions, sharing some similarities with human ONJ findings, such as reduced amount of TRAP + osteoclasts, and increased leukocyte infiltration compared to control animals, confirming the disruption of inflammatory response and osteoclasts resorption by nBPs during the alveolar bone healing^25^.

Considering inflammatory candidates to play a role in ONJ development, lipid signaling mediators derived from arachidonic acid might be considered as key players, since these pathways have been shown in bone healing and metabolism^29^. Both cyclooxygenase-2 (COX-2) and 5-lypoxigenase (5-LO) have been identified in specific sites during bone repair^30^, and are highly expressed in macrophages^31^. COX-2 products (specifically PGE2) appears to be produced by osteocytes and protect these cells from apoptosis^32^. Furthermore, PGE2 are considered beneficial for bone healing^33,34^, while 5-LO and its products (i.e.LTB4 and the CysLTs), seems to exert a detrimental effect on this process, inducing osteoclastogenesis^29,35,36^. Mutant mice for 5-LO enzyme (5LOKO) show accelerated fractured healing^37^, and aged female 5LOKO mice also present improved bone regeneration in femur defects^29^. These previous findings indicate that inhibition of 5LO seems to favor bone healing in endochondral bones of young and aged mice. Also, previous evidence show that 5LO inhibition results in an osteopetrotic-like skeletal condition with few osteoclasts and, consequently, low bone turnover in these animals^20,36^. Such as nBPs, it appears that absence of 5LO improves bone quality of endochondral bones. Other studies using 129 Sv background strain show that 5LOKO mice present decreased bone resorption in oral cavity during the mechanical loading^38^. However, the effects of 5LO inhibition in intramembranous bone healing post tooth extraction remains to be explored, as well in a model of compromised healing such as ONJ in aged animals. The primary objective of this study was to analyze the role of 5LO on skeletal phenotype and intramembranous bone healing with or without ZL therapy. The secondary objective was comparatively analyze the ONJ-development post tooth extraction in WT and 5LOKO aged female mice by a cause-and-effect manner.

## Results

### Surgical outcomes

Due to the *osteopetrotic*-like bone in elderly females, two 5LOKO animals (1 control and 1 ZL) were lost during tooth extraction because of surgical complications. The microscopic analysis were carried out with 4 animals for 7 days time point in these groups. All animals presented healed oral mucosa at 21 days and no post-surgical clinical complications (i.e.purulent secretion, loss of weight) were observed.

### Effects of ZL administration on skeletal phenotyping WT and 5LOKO aged female mice

**Figure 1:**
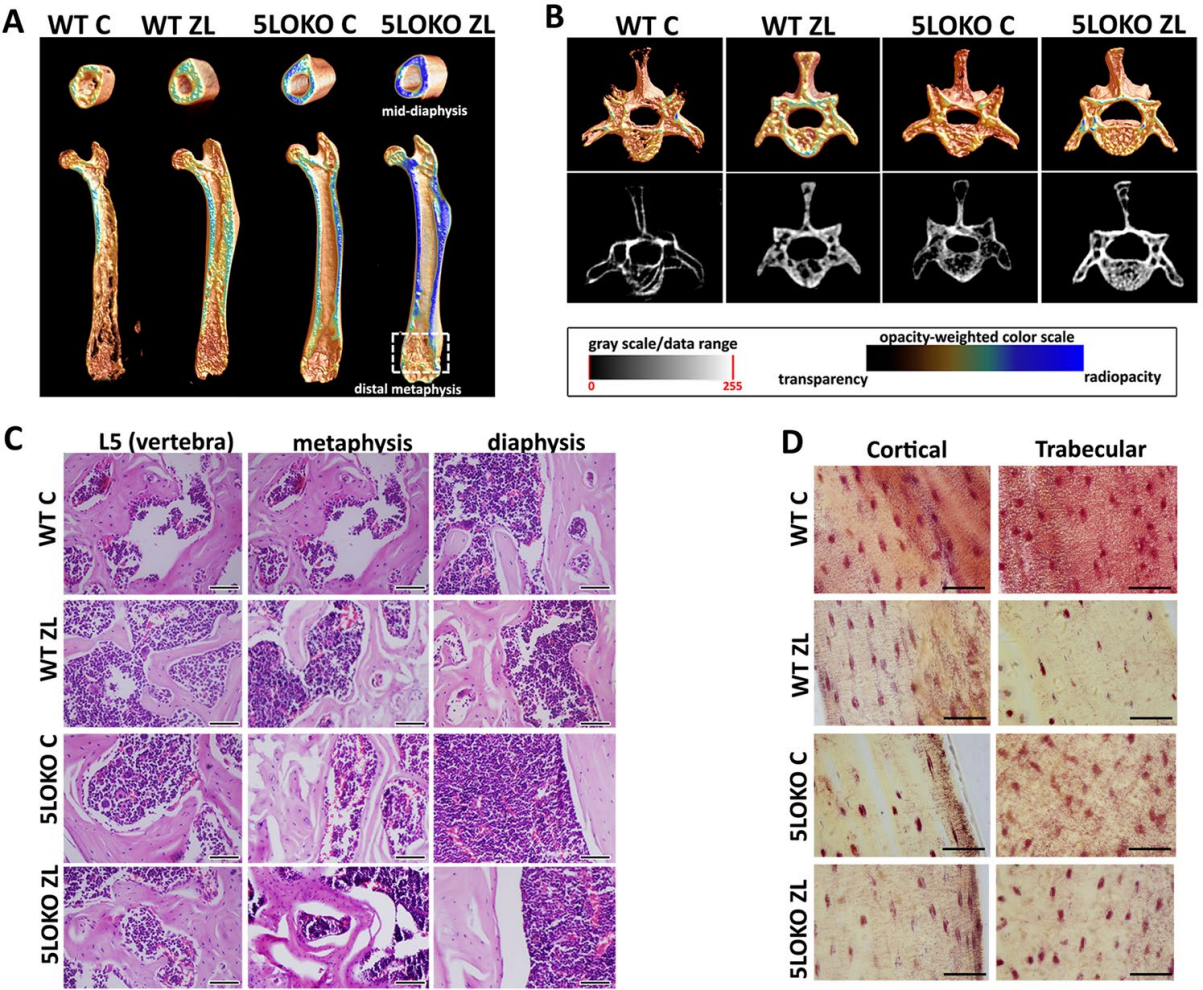
**Qualitative results of microCT and histological analysis for WT vs. 5LOKO mice treated or not with ZL on skeletal phenotyping WT and 5LOKO aged female mice. **Aged 129 Sv WT and 5LOKO female mice were treated with 0.9% saline solution (Vehice) or ZL (250ug/Kg), once a week, for 7 weeks. Mice were euthanized and femur and L5 vertebrae were collected for analysis by microCT (**A**), H&E (**B**) and Picro-thionin (Schmorl) staining for osteocytes lacunae (**C**). (**C**) Scale bar 100 µm; (**D**) scale bar 50 µm. (**A**) Three-dimensional representative images obtained with the CT-Vox software (Skyscan, Kontich, Belgium).

Medial diaphysis of femur showed increased hyperdensity in 5LOKO controls compared to the WT control or WT ZL ones. This increase was confirmed by the differences obtained from the analysis of cortical area (Ct.Ar), observed between WT C (1.808 ± 0.37) vs. WT ZL (2.692 ± 0.66) and WT C vs. 5LOKO C (2.87 ± 1.05) (Fig. 1A and Table 1). Significant differences were also found in distal metaphysis of the femur considering BV/TV between WT C (10.77 ± 2.69) vs. WT ZL (30.9 ± 7.74), WT C vs. 5LOKO C (19.32 ± 4.93), and WT C vs. 5LOKO ZL (22.17 ± 2.71). WT-ZL presented a significant improvement in Tb.Th when compared to all groups (Table 1). Decreased Tb.Sp was detected in WT ZL (0.62 ± 0.17) and 5LOKO C (0.70 ± 0.07) in comparison to WT C (1.09 ± 0.16) (Fig. 1B and Table 1). For L5 vertebra, significant differences were observed in trabecular body BV/TV when comparing WT C (23.12 ± 5) vs. WT ZL (47.37 ± 23.47), vs. 5LOKO C (49.6 ± 9.36), and vs. 5LOKO ZL (49.92 ± 12.00). Also, Tb.Th parameter was significantly increased in WT ZL (0.27 ± 0.22) in comparison to the WT-ZL (0.12 ± 0.03) (Fig. 1 and Table 1). In the femur histopathological analysis, WT C group showed highly cellularized trabecular bone. The other groups presented mature bone trabeculae, with fewer osteocytes and no morphological signs of remodeling. A particular aspect of the hematopoietic tissue was evident in ZL groups, which was its intense cell contingent in the metaphysis. For L5 vertebra, WT groups (controls and ZL) presented L5 vertebra with a thin remodeling cortical bone, and highly cellularized trabecular bone surrounded by hematopoietic tissue. 5LOKO and WT ZL groups presented mature, but irregular, bone trabeculae. 5LOKO and ZL treated mice showed fully cellularized hematopoietic tissue (Fig. 1C). Quantitative analyses by picro-thionin (Schmorl) staining revealed significant decrease of the osteocytes’ lacunae of WT ZL (2.91 ± 1.58) *vs.* WT control (10.43 ± 1.51) femurs (Fig. 1D, Table 1).

**Table 1:** Quantitative results of microCT and histological analysis for skeletal phenotyping in WT *vs.* 5LOKO mice treated or not with ZL.

Parameters	Sites/groups	WT C	WT ZL	5LOKO C	5LOKO ZL
BV/TV (mm^3^)	Femur diaphysis	1.808 ± 0.37*^#^	2.69 ± 0.66^*#*^	2.87 ± 1.05*	2.58 ± 0.38
BV/TV (%)	Femur diaphysis	14.21 ± 1.72	24.81 ± 6.23	18.11 ± 5.01	25.98 ± 3.02
	Femur metaphysis	10.77 ± 2.69*^#&^	30.9 ± 7.74^*#*^	19.32 ± 4.93*	22.17 ± 2.71^&^
	L5 vertebrae	23.12 ± 5*^#&^	47.37 ± 23.47^*#*^	49.6 ± 9.36*	49.92 ± 12.00^&^
Tb.Th (mm)	Femur metaphysis	0.17 ± 0.02*^#&^	0.36 ± 0.04^*#*^	0.20 ± 0.02*^#^	0.28 ± 0.03^&*#*^
	L5 vertebrae	0.12 ± 0.03*^#&^	0.27 ± 0.22^*#*^	0.20 ± 0.04*	0.25 ± 0.06^&^
Tb.Sp (mm)	Femur metaphysis	1.09 ± 0.16*^#&^	0.62 ± 0.17^*#*^	0.70 ± 0.07*	0.75 ± 0.12^&^
Tb.N (1/mm)	Femur metaphysis	0.53 ± 0.17	0.83 ± 0.31	0.83 ± 0.28	0.76 ± 0.12
	L5 vertebrae	1.21 ± 0.40	1.86 ± 0.28	2.17 ± 0.27	2.27 ± 0.31
# osteocytes’	Cortical	8.00 ± 3.52	6.18 ± 2.82	5.14 ± 3.02	6.85 ± 2.47
lacunae	Trabecular	10.43 ± 1.51^*#*^	2.09 ± 1.57^*#*^	6.20 ± 2.68	5.57 ± 1.71

Results are presented as the means (± SD) for each parameter.

In the comparison between columns, symbol* indicates comparison between the effect of 5LO knockout vs. WT; symbol^#^ indicates the effect of ZL treatment in groups of same genotypes (WT vs. WT ZL); and symbol^&^ indicates the effect of ZL treatment in different genotypes (WT vs. 5LOKO). Statistically significant differences are indicated between groups with equal symbols (p < 0.05).

### ZL decreased quantity and quality or inorganic matrix in alveolar sockets of WT mice, but not in 5LOKO mice

**Figure 2:**
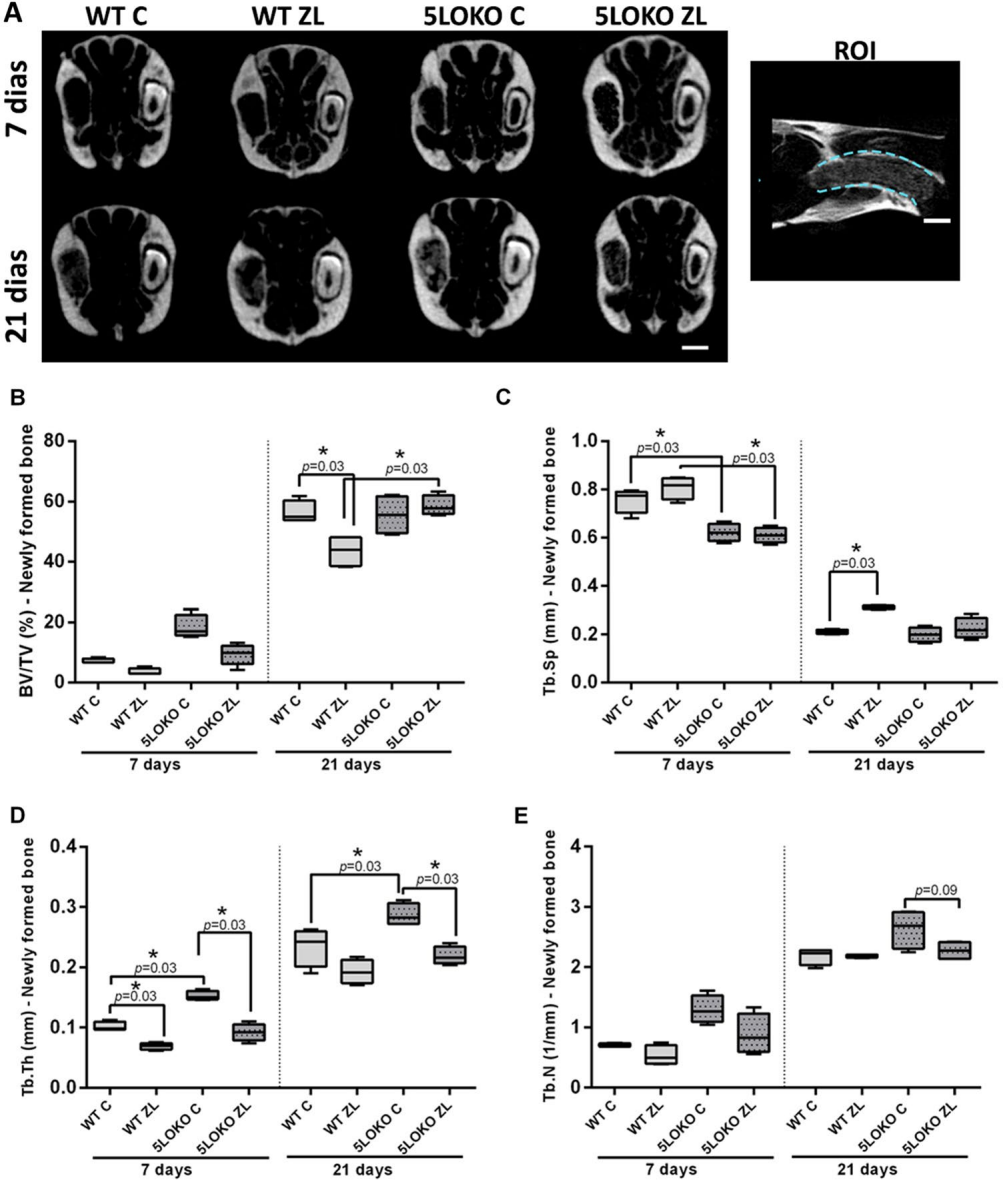
**MicroCT analysis of alveolar sockets post tooth extraction from WT vs. 5LOKO mice treated or not with ZL.** Aged 129 Sv WT and 5LOKO female mice received IP injections of 0.9% saline solution (C) or 250 μg/Kg (ZL groups) once a week, and upper right incisor were extracted at 4 week of each treatment. Mice were euthanized for maxillary bones removal after 7 days and 21 days post tooth extraction. (**A**) Representative transaxial images of bone hyperdensity. Scale bar 1.5 mm. (**B**) Bone volume/Tissue Volume (BV/TV, %), (**C**) Trabecular Separation (Tb.Sp), (**D**) Trabecular Thickness (Tb.Th), and (**E**) Trabecular Number (Tb.N) for new bone in alveolar sockets and 7 and 21 days post tooth extraction. Quantitative results are presented with Box and whiskers (Min to Max). Symbol * indicates a statistically significant differences between different treatments and/or genotypes (p < 0.05).

We used microCT imaging to investigate bone microarchitecture in alveolar bone repair post tooth extraction in aged female. This analysis made possible the evaluation of the proportion between the newly formed bone and total tissue (BV/TV,%), and the quality of the trabecular bone (Tb.Th, Tb.Sp, Tb.N). After 21 days, significant increased BV/TV was observed when comparing WT C (56.34 ± 3.8%) *vs*. WT-ZL (43.55 ± 5.27%), and WT-ZL vs. 5LOKO-ZL (43.55 ± 5.27%vs 58.55 ± 3.37%). No differences were detected in the comparison between WT C vs 5LOKO C (56.34 ± 3.8% vs 55.58 ± 6.64%), and 5LOKO ZL. 5LOKO ZL (Fig. 2). Tb.Sp was significantly increased in WT ZL (0.81 ± 0.04) when compared to WT C (0.76 ± 0.05), and 5LOKO C (0.62 ± 0.04) or ZL (0.61 ± 0.03) at day 7. At day 21, significant differences were observed between WT C (0.21 ± 0.01) and WT ZL (0.31 ± 0.01), with an increased Tb.SP observed in WT ZL animals. At day 7 Tb.Th was significantly increased in WT and 5LOKO control groups, in comparison to their matched ZL groups. At day 21, 5LOKO C (0.28 ± 0.02) presented increased Tb.Th when compared to WT C (0.23 ± 0.03) and 5LOKO ZL (0.21 ± 0.02). (Fig. 2). No significant differences were detected among the groups considering Tb.N (Fig. 2).

### ZL induced ONJ-like lesion features in WT mice vs. delayed bone healing in 5LOKO mice

**Figure 3:**
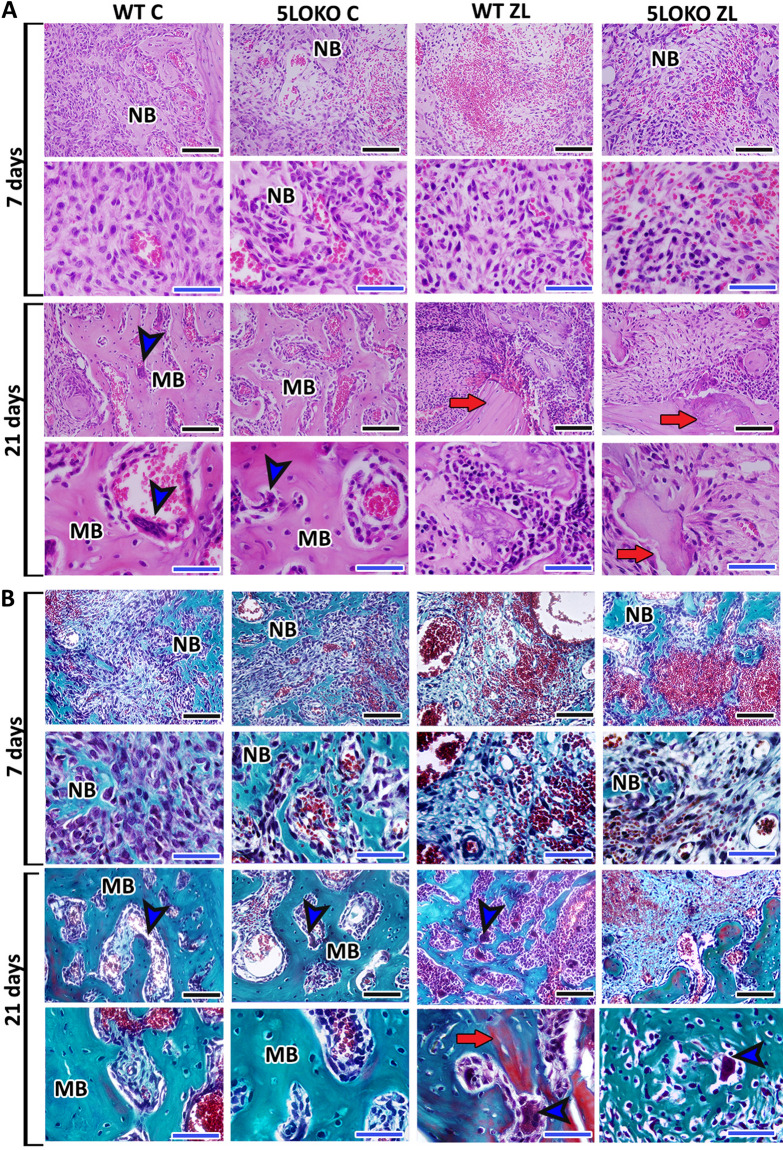
**Histopathological analysis of alveolar sockets post tooth extraction from WT vs. 5LOKO mice treated or not with ZL.** Aged 129 Sv WT and 5LOKO female mice received IP injections of 0.9% saline solution (**C**) or 250 μg/Kg (ZL groups) once a week, and upper right incisor were extracted at 4 weeks of each treatment. Mice were euthanized for maxillary bones removal after 7 days and 21 days post tooth extraction. (**A**) Representative transversal sections are observed throughout days 7 and 21, from the central area of alveolar sockets. Histological slides were stained with HE (A) and modified Goldner trichrome + Alcian blue (**B**). Images were captured at 10 × and 100 × magnification (panels). Black scale bar = 100 µm, Blue scale bar 50 µm. *NB* New bone formation, *BC* Blood clot, *MB* mature remodeling trabeculae, blue arrowheads = osteoclasts; red arrows = non-viable old bone.

**Figure 4:**
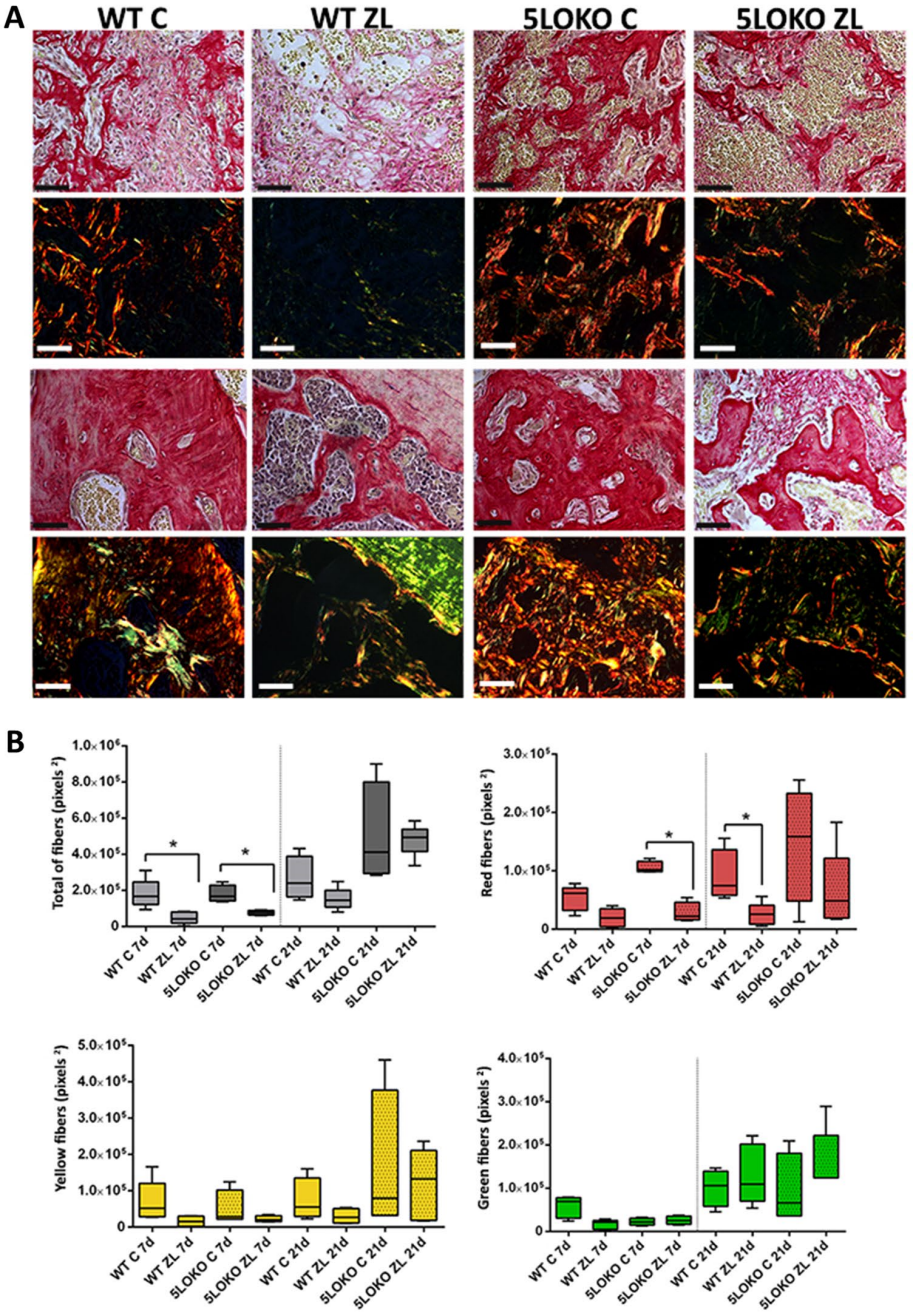
**Birefringence analysis of collagen fibers in alveolar sockets post tooth extraction from WT vs. 5LOKO mice treated or not with ZL.** Aged 129 Sv WT and 5LOKO female mice received IP injections of 0.9% saline solution (**C**) or 250 μg/Kg (ZL groups) once a week, and upper right incisor were extracted at 4 weeks of each treatment. Mice were euthanized for maxillary bones removal after 7 days and 21 days post tooth extraction. (**A**) Representative transversal sections alveolar socket upon polarized and conventional light. Polarized light shows green birefringence color for thinner collagen fibers; yellow and red colors at birefringence analysis indicate thick collagen fibers. Original magnification was 40x, Scale bar: 100 µm. (**B**) Total area (pixels^2^) of collagen and area for each birefringence color (pixels^2^) (green, yellow and red). RGB values for green spectra (R:114–255, G:78–255, B:10–255), yellow (R:196–255,G:32–255,B:10–255) and red (R:162–255,G:0–175,B:0–255). Quantitative results are presented with Box and whiskers (Min to Max). Symbol * indicates a statistically significant differences between different treatments and/or genotypes (p < 0.05).

HE was used for histopathological evaluation of oral mucosa, which was observed completely healed in all groups and time points (Supplementary Fig. 2). HE and modified Goldner trichrome + Alcian Blue were used for histopathological and histomorphometric evaluation of alveolar sockets post tooth extraction (Fig. 3A,B). WT C presented a suitable and discrete osteogenic activity at day 7 that evolved to the formation of mature remodeling trabeculae at day 21. On the other side, 5LOKO control animals presented higher osteogenic activity with bone matrix production compared to WT C at day 7 (29.71 ± 4.96 vs. 21.56 ± 7.91). Reduced bone matrix was observed in WT-ZL as compared to all groups. No significant difference in bone matrix were observed at day 21 between 5LOKO C (45.67 ± 12.05) and WT C (37.24 ± 3.24). Qualitatively, 5LOKO C presented mature bone trabeculae, with organized medullary spaces presenting focal mononuclear infiltration (Fig. 3A,B). ZL administration in WT animals caused ONJ-like lesions features, with the presence of residual blood clot amongst granulation tissue at day 7. At day 21, WT-ZL mice presented irregular bone trabeculae, with large lacunae with no osteocytes, amongst a persistent granulation tissue infiltrate by mononuclear leukocytes and bone sequestrum. In histomorphometry analysis, the number of osteocytes were found to be significantly reduced in WT ZL (1.06 ± 1.33) compared to WT C (6.51 ± 2.06), and also reduced in 5LOKO C (2.95 ± 0.77) and 5LOKO ZL (1.09 ± 0.83). However, empty lacunae (a parameter characteristic of ONJ-like lesions), was significantly higher in WT ZL (7.43 ± 1.85) compared to all other groups. Finally, at 21 days, WT ZL presented a persistent inflammatory infiltrate (18.33 ± 6.03), with significant differences compared to WT C (1.62 ± 0.62) and 5LOKO C (3.62 ± 1.37). The inflammatory infiltrate in 5LOKO ZL (10.01 ± 1.51) was also increased as compared to 5LOKO controls (3.62 ± 1.37) (Table 2). Large and round osteoclasts were also observed inside the sockets, non-adhered to bone surface in WT-ZL. Effects of ZL on 5LOKO mice were distinct especially when analyzing day 21, when osteogenic activity with newly bone formation alternated by the presence of non-viable bone tissue and eventual bone sequestrum was observed, as demonstrated in Fig. 3. In the birefringence analysis for collagen content of newly formed bone, a significant increase of total collagen fibers was observed in WT C (181,054 ± 79,366 pixels^2^) in comparison to WT ZL (47,876 ± 33,104 pixels^2^), as well as in the comparison between 5LOKO C (179,049 ± 478,95 pixels^2^) and 5LOKO ZL (76,483 ± 13,269 pixels^2^) at day 7. No differences were detected among the groups at day 21. Red spectra fibers were increased in 5LOKO controls (105,761 ± 103,69) at day 7 in comparison to 5LOKO ZL (28,161 ± 17,584). At day 21 WT C showed increased number of red fibers (89,764 ± 45,234) compared to WT ZL (26,553 ± 18,534) as shown in Fig. 4.

**Table 2:** Histomorphometry for alveolar bone healing in WT *vs.* 5LOKO aged female mice treated or not with ZL.

Parameters	Period	WT C	WT ZL	5LOKO C	5LOKO ZL
Inflammatory Infiltrate	7 days	5.53 ± 1.17	14.36 ± 3.93	4.79 ± 1.59	7.67 ± 2.43
	21 days	1.62 ± 0.62^*#*^	18.33 ± 6.03^*#*&^	3.62 ± 1.37^*#*&^	10.01 ± 1.51^*#*^
Fibers and Fibroblasts	7 days	24.17 ± 5.89	19.32 ± 12.91	15.60 ± 9.81	23.26 ± 9.41
	21 days	11.11 ± 4.12	16.88 ± 3.06	10.74 ± 5.9	22.20 ± 11.12
Bone Matrix	7 days	21.56 ± 7.91^*#*^	7.59 ± 2.11^*#*&^	29.71 ± 4.96^*#*&^	18.75 ± 3.5^*#*&^
	21 days	37.24 ± 3.24^*#*^	18.83 ± 12.51^*#*^	45.67 ± 12.05^*#*^	31.18 ± 5.15^*#*^
Osteocytes	7 days	6.51 ± 2.06*^#^	1.06 ± 1.33^*#*&^	2.95 ± 0.77*^#&^	1.09 ± 0.83^*#*^
	21 days	5.59 ± 3.0024	2.35 ± 2.30	5.75 ± 1.89	3.98 ± 1.24
Empty Lacunae	7 days	0.025 ± .0.05	1.50 ± 1.29	0.03 ± 0.05	1.00 ± 0.81
	21 days	0.04 ± 0.08^*#*^	7.43 ± 1.85^*#*^	1.10 ± 0.74	3.05 ± 1.76

Results are presented as the means (± SD) for each parameter.

In the comparison between columns, symbol* indicates comparison between the effect of 5LO knockout vs. WT; symbol^#^ indicates the effect of ZL treatment in groups of same genotypes (WT vs. WT ZL); and symbol^&^ indicates the effect of ZL treatment in different genotypes (i.e. WT-ZL vs. 5LOKO-ZL or WT-ZL vs. 5LOKO C ). Statistically significant differences are indicated between groups with equal symbols (p < 0.05).

### 5LO is highly expressed in ONJ-like lesions in comparison to healed alveolar sockets

**Figure 5:**
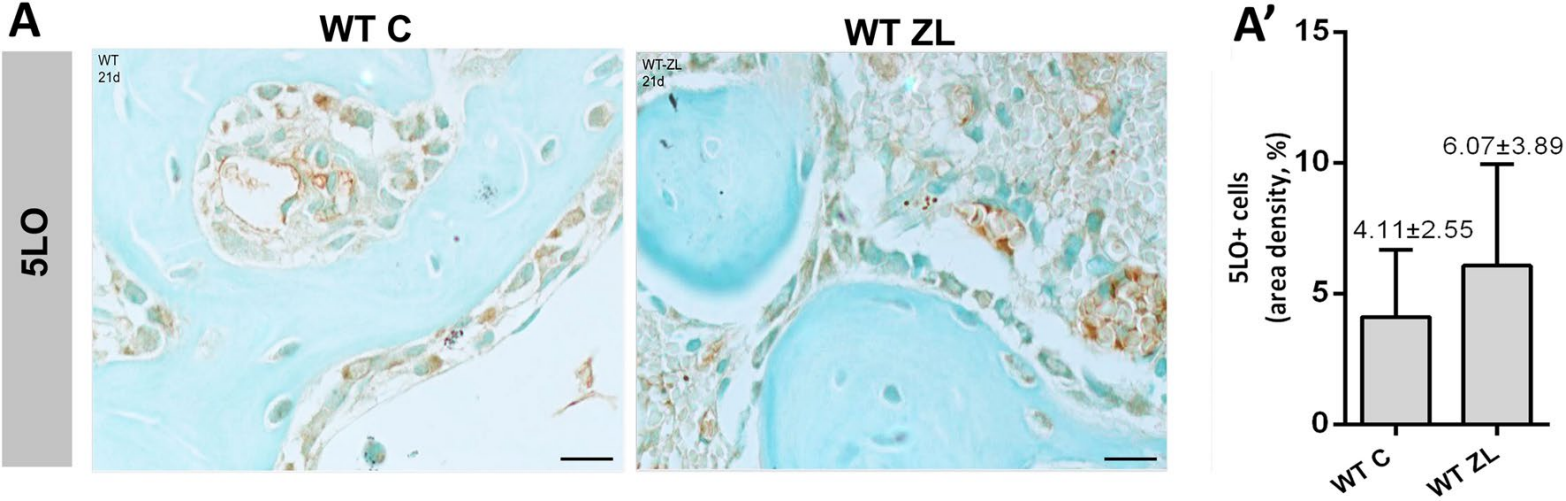
**Immunolabeling and quantification of 5LO + cells at 21 days post tooth extraction in WT C vs. WT ZL.** Aged 129 Sv WT female mice received IP injections of 0.9% saline solution (**C**) or 250 μg/Kg (ZL groups) once a week, and upper right incisor were extracted at 4 week of each treatment. (**A**) Representative transversal sections from the central area of alveolar sockets stained for 5LO + cells in WT C and WT ZL group. (**A’**) Quantitative and comparative analysis of detached 5LO + cells in WT C vs. Scale bar 30 µm. WT ZL mice at 21 days post-extraction. Results are presented as the means (± SD) of area density (%). Symbol * indicates a statistically significant difference between groups (p < 0.05). DAB chromogen and counterstained with Fast green.

We performed immunohistochemistry for 5LO positive cells (5LO +) in alveolar sockets of WT C vs WT ZL mice at 21 days. As observed in the Fig. 5, 5LO was detected in osteoblasts, lining cells, osteocytes, and fibroblasts, in both WT C and WT ZL groups. In osteoblasts, 5LO labeling was observed in the cytosol and perinuclear simultaneously (Fig. 5), and as they were being incorporated to the bone matrix, perinuclear labeling was more evident. Despite a slightly higher area density in 5LO + cells was noted in WT ZL (6.07 ± 3.39) group *vs*. WT C (4.11 ± 2.55), no significant differences were detected between these groups. WT ZL also presented inflammatory cells with positive immunolabeling for 5LO, while in controls this expression was predominant in bone cells. No 5LO + osteoclasts were observed.

### 5LO knockout leaded to enhanced expression of COX2 and osteogenic markers during alveolar socket healing in aged female mice

**Figure 6:**
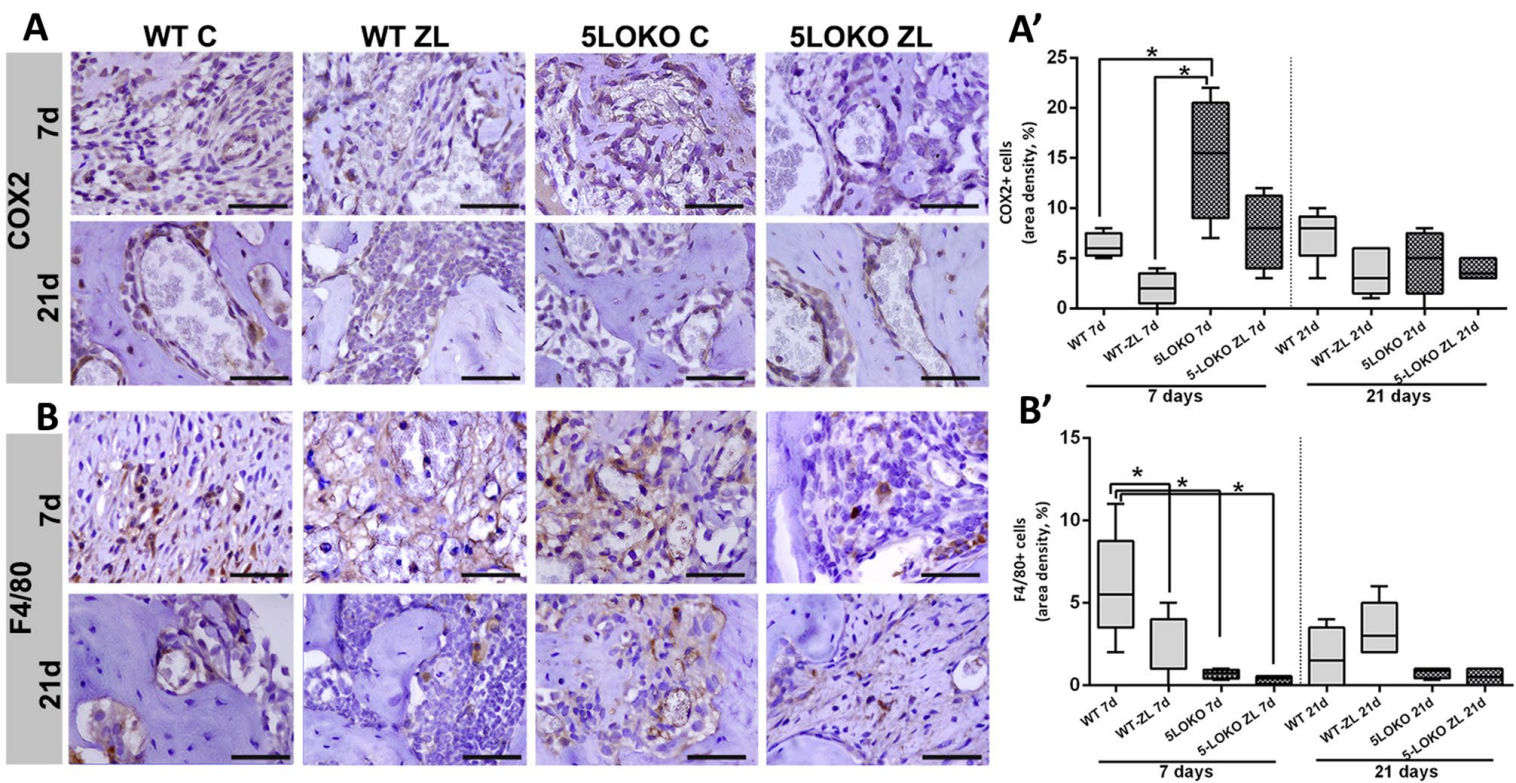
**Immunolabeling and quantification of COX2 and F4/80 + cells at 7 and 21 days post tooth extraction in WT and 5LOKO mice, treated or not with ZL.** Aged 129 Sv WT female mice received IP injections of 0.9% saline solution (**C**) or 250 μg/Kg (ZL groups) once a week, and upper right incisor were extracted at 4 week of each treatment. Mice were euthanized for maxillary bones removal after 7 days and 21 days post tooth extraction. (**A**) Representative transversal sections from the central area of alveolar sockets stained for COX2 + cells (**A**) and F4/0 + cells (**B**). Quantitative and comparative analysis of COX2 + cells (**A’**) and F4/80 + cells (**B’**) among groups. Scale bar: 50 µm. Quantitative results are presented with Box and whiskers (Min to Max). Symbol * indicates a statistically significant difference between groups (p < 0.05). DAB chromogen and counterstaining with Harris’ Hematoxylin.

**Figure 7:**
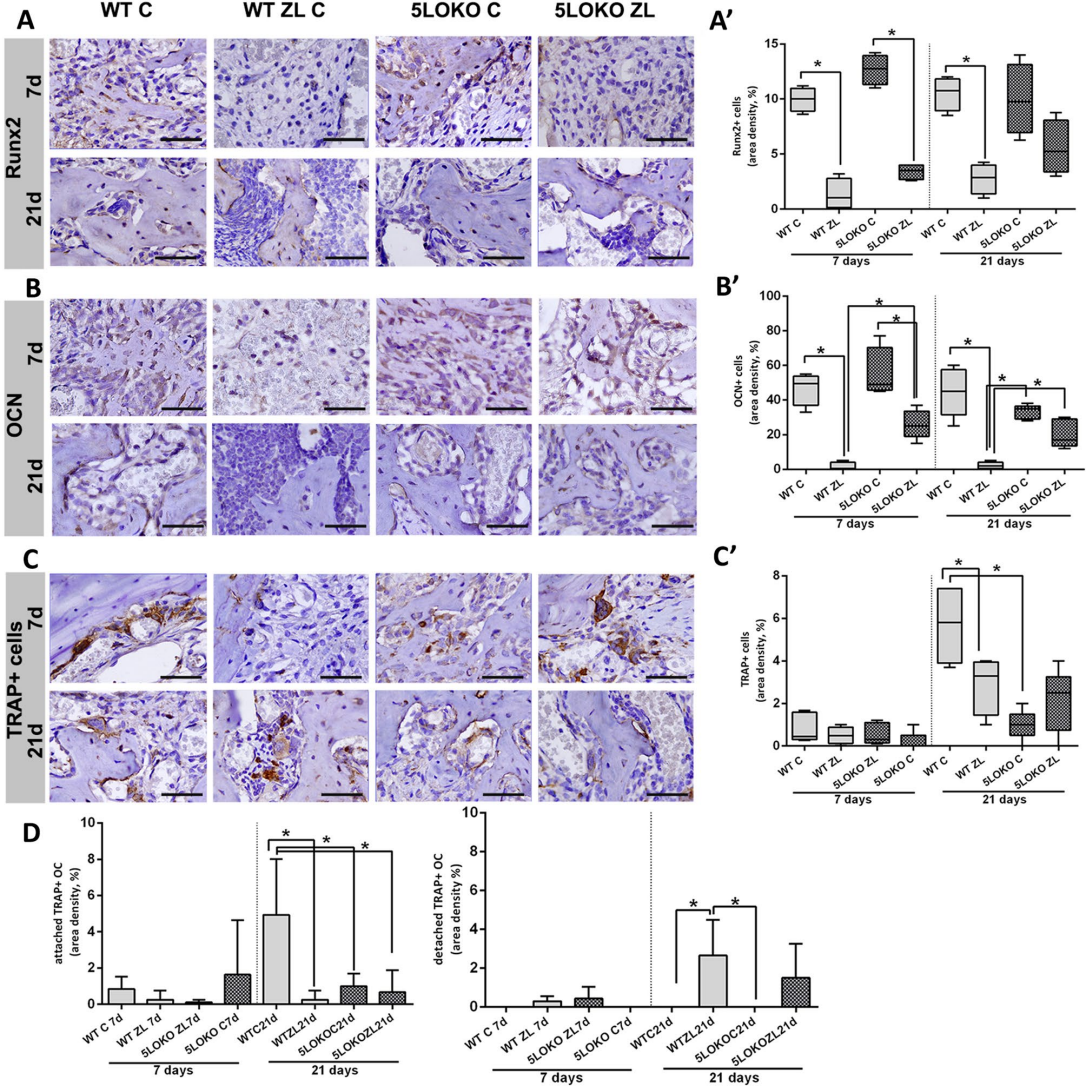
**Immunolabeling and quantification for Runx2 + , OCN + and TRAP + cells at 7 and 21 days post tooth extraction in WT and 5LOKO mice, treated or not with ZL. **Aged 129 Sv WT female mice received IP injections of 0.9% saline solution (**C**) or 250 μg/Kg (ZL groups) once a week, and upper right incisor were extracted at 4 week of each treatment. Mice were euthanized for maxillary bones removal after 7 days and 21 days post tooth extraction. (**A**) Representative transversal sections from the central area of alveolar sockets stained for Runx2 + cells (**A**), OCN + cells (**B**) and TRAP + cells (**C**), Scale bar: 50 µm. Quantitative and comparative analysis of Runx2 + cells (**A’**), OCN + cells (**B’**), and TRAP + cells (**C’**) among groups. Quantitative results are presented with Box and whiskers (Min to Max). (**D**,**E**) Quantitative analysis for attached and detached TRAP + cells. Results are presented as the means (± SD) of area density (%). Symbol * indicates a statistically significant difference between groups (p < 0.05). DAB chromogen and counterstaining with Harris’ Hematoxylin.

Last, we performed immunohistochemistry for COX2 + cells, F4/80 + macrophages and bone markers (TRAP, Runx2, OCN) for all groups and time points. After 7 days of socket healing, COX-2 was predominantly expressed in the osteoblasts in both WT and 5LOKO controls mice (Fig. 6). Quantitative analysis indicated that COX-2 + cells were increased in 5LOKO control group (15.00 ± 6.16) as compared to WT C (6.25 ± 1.26) and WT ZL (2.00 ± 1.58) at 7 days. No differences were found at 21 days. F4/80 + macrophages were observed in earlier (7 days) and later (21 days) of healing (Fig. 6), with increased area density in WT C (6 ± 3,162) *vs*. WT ZL (1.90 ± 1.71), and *vs*. 5LOKO C (0.70 ± 0.27) or 5LOKO ZL (0.35 ± 0.25) at day 7. No differences were detected at day 21. Considering bone markers, a significant increase of Runx2 + cells was observed in WT C (5.68 ± 1.58) when compared to WT ZL (2.90 ± 1.37) (Fig. 7). Runx2 + cells were significantly increased in 5LOKO ZL (2.17 ± 1.47) when compared to 5LOKO controls (1.00 ± 0.71) at day 7. At day 21, a significant difference in area density of Runx2 + cells were observed in WT C (10.50 ± 1.51) vs. WT ZL (2.75 ± 1.37) groups. As a later marker of bone differentiation, OCN immunolabeling was maintained elevated in all controls in comparison to their respective ZL groups. At day 7 days, WT ZL group presented significant decrease in OCN + cells (1.50 ± 2.38) when compared to WT C (46.75 ± 9.53), 5LOKO C (55.00 ± 14.81) and 5LOKO ZL (26.00 ± 8.51) (Fig. 7B,B’). With a delayed healing, 5LOKO ZL presented significantly reduced numbers of OCN + osteoblasts as compared to 5LOKO C. At 21 days, OCN was also found significantly reduced mainly in WT ZL groups (2.20 ± 1.92) in comparison to WT Control (44.60 ± 13.90), 5LOKO C (33.20 ± 4.08) and 5LOKO ZL (20.40 ± 8.08), suggesting a more relevant effect of ZL in bone formation and maturing in WT animals. Lastly, considering total of TRAP + cells (Fig. 7C,C’), significant differences were detected when comparing WT C group (5.68 ± 1.575) vs. WT ZL (2.9 ± 1.371) and vs. 5LOKO C (1 ± 0.7071), at day 21. Because several osteoclasts could be seen detached from bone surface in ZL groups, we also analyzed the number of detached and attached TRAP + osteoclasts (Fig. 7D,E). Considering only TRAP + cells attached to bone surface, a significant decrease was detected in WT ZL (0.25 ± 050), in 5LOKO C (1.00 ± 0.70) and in 5LOKO ZL (0.66 ± 1.21) in comparison to WT C (4.94 ± 3.08) at 21 days. On the other hand, a significant increase of TRAP + non-attached cells in WT ZL (2.65 ± 1.84) animals in comparison to WT C (0.00 ± 0.00), 5LOKO C (2.00 ± 4.47) and 5LOKO ZL (1.50 ± 1.76) (Fig. 7).

## Discussion

Increasing age, female gender and intravenous formulations of nBPs appear as important risk factors for development of ONJ^24^. Several studies have evaluated ONJ-like lesions in rodents, applying oral nBPs, such as alendronate^39,40^ or parenteral nBPs, such as ZL^4,5^. Because parenteral administration of ZL is prescribed for treating bone damage in different conditions, such as severe age-related osteoporosis^4,5^, glucocorticoid induced bone loss^41^ and bone resorption induced by metastasis^42^, this was the medication of choice in this study. The detrimental effects of ZL on dental socket healing process and development of ONJ-like lesions in aged female 129 Sv/Ev mice have been demonstrated in a previous study designed by our group^25^ due to the lack of pre-clinical investigations that considered the variables gender and age at a time. In fact, most of the studies in this field are performed in young or middle aged animals, which are useful for understanding the impact of nBP on young/adult skeleton and craniofacial bones. In the present study, we applied this experimental model in aged female mice to investigate the influence of 5LO on dental socket healing process and ONJ-like lesions development^35,36^ Studies on ONJ pathogeny refers to the action of ZL on osteoclasts as the main mechanism leading to the ONJ development^11,14^. Continuous use of nBPs leads to a disturbance in osteoclasts’ resorption capacity and result in low of ability to recover a damaged bone^11^. In fact, our previous model of ONJ-like lesions in aged females 129WT mice, clearly demonstrated a disturbance in healing response in alveolar sockets at 21 days, with reduced amount of TRAP + osteoclasts in alveolar sockets of mice treated with ZL^25^. Based on this previous evidence in ONJ pathogeny, and the findings on bone phenotype of 5LOKO mice, our initial hypothesis was that 5LOKO animals were more prompt to develop ONJ-like lesions in a model of tooth extraction in 5LOKO C (with no medication). Mutant mice for 5LO might present lower amount of osteoclasts and similar bone microarchitecture compared to WT ZL group, although, these differences resulted from distinct biological mechanisms^43^. Aged WT female mice appeared to decrease bone mass after 17 months compared to young controls, while aged 5LOKO mice presented a more robust phenotype with no major changes compared to 5LOKO young controls ^29^. Since 5LO inhibition results in an osteopetrotic-like skeletal condition with few osteoclasts, consequently, the low bone turnover may contribute to the maintenance of their bone mass during their lives^36^ This characteristic was the key point for the selection of this animal model for the present investigation. In the microCT analysis for skeletal phenotyping, it was observed that 5LOKO control mice showed femur cortical BV/TV comparable to the WT mice treated with ZL (Fig. 1).

We evaluated if 5LO knockout condition could cause ONJ-like lesions in elderly female mice. Interestingly, no evidence of ONJ-like lesions was found in histopathological analysis of 5LOKO controls. On the contrary, 5LOKO mice presented accelerated bone formation when compared to WT C at 7 days. While osteogenesis and new bone deposition occurred predominantly in the bone walls from dental sockets of WT C, new osteogenesis was observed in the entire socket of 5LOKO controls, also coherent with presence of Runx2 + cells at this period. It has been previously demonstrated that animals lacking 5LO have higher amount COX derivates (i.e. PGF2α) and basal concentrations of PGE2 (comparable to C) in fracture healing sites^37^. Our previous studies using a model of bone and muscle healing in different demographics of 129 Sv WT and 5LOKO mice, showed a higher amount of PGE2 in muscles of 5LOKO aged female mice compared to aged WT C^29^. It is reasonable to postulate that improved bone healing observed in aged 5LOKO controls could be associate with a predominant availability of COX2 in the initial phase of bone repair compared to WT C. Indeed, increased area density of COX2 + cells was also detected in 5LOKO group when compared to WT C at 7 days. While the capacity of early bone formation of aged 5LOKO females have already been described by our previous study on endochondral bone repair model (femur)^29^, the findings from the present study reveals the role for 5LO on intramembranous bone repair (maxillary dental socket). Dental sockets are composed by predominantly trabecular bone, and are exposed to the oral cavity, with more challenging conditions for healing than closed defects in long bones.

Next, a group of WT and 5LOKO were treated with ZL and subjected to tooth extraction. Results from microCT and histological analysis indicated an attenuated development of ONJ-lesions which could be rather than a delayed bone healing in 5LOKO ZL groups. Of note, when ZL was administered to the WT and 5LOKO animals, some histological alterations were seen in both group genotypes, with increased severity in WT mice. Despite a complete healing of oral mucosa in all animals with ONJ-like lesions, the underlying bone presented important features of non-viable bone, as observed in stage 0 of ONJ in humans^44^. The observed microtomographic and histopathological features of ONJ-like lesions in the WT mice were compatible to the a previous study using this model^25^. The microCT images clearly indicated that ZL significantly influenced inorganic bone matrix deposition and the quality of microarchitecture on dental sockets of WT and 5LOKO aged female mice, a more severe disruption was observed in WT ZL mice (Fig. 2).

Since 5LOKO controls mice presented an attenuated inflammatory response compared to WT C, we assumed that modulation of inflammatory response caused by 5LO deletion could be a factor to improve and accelerate the bone formation in 5LOKO mice, as well to attenuate the ONJ-lesions in 5LOKO ZL group. It has been shown that ONJ development is related to a disturbed inflammatory response induced by nBPs^45^. Also, monocytes/macrophages seems to play a crucial role on the pathogenesis of these lesions, as well the imbalanced polarization towards M1 inflammatory phenotypes^22,46,47^. The immunohistochemistry analysis for 5LO in WT sockets shed light on 5LO immunolabeling pattern on healed sockets and ONJ-like lesions, in which 5LO could be seen in osteocytes, osteoblasts, lining cells in both WT and WT ZL groups at day 21. A slight, but no significant increase (p = 0.05) in 5LO + cells was detected in WT ZL as compared to WT C, especially in inflammatory cells. These findings indicated that 5LO may play a role on bone remodeling and maturation, but also on disturbed inflammatory and healing response in ONJ-like lesions. Interestingly, WT ZL showed characteristic features of ONJ-like lesions associated to an intense inflammation at day 21, as observed in histomorphometry (Table 2) and increased area density of 5LO compared to WT C (Fig. 5). On the other hand, 5LOKO ZL mice presented mild inflammation, with maturing bone trabeculae in the same evaluation period compared to WT ZL. Intriguingly, a higher number of F4/80 + macrophages were detected in WT control when compared to WT ZL and 5LOKO controls at day 7. At this point, it is important to emphasize that future studies are necessary to understand the polarization status of macrophages in these conditions, which may explain how decreased amount of macrophages in WT ZL may sustain a higher inflammation in these animals, while WT C progress for healing. Also, TRAP + cells presented the same proportion of F4/80 + cells that strength their common origin, which may reflect in the late stages of bone repair^16^. Of note, usually animals treated with ZL developed typical features of ONJ-like lesion in rodents, with reduced amount of TRAP + osteoclasts, and increased leukocyte infiltration compared to control animals, confirming the disruption of inflammatory response and osteoclasts resorption by nBPs^25^. In agreement to these findings, it has been shown that inhibition of 5LO or its products may contribute to attenuate inflammatory response in a mouse model of of LPS-induced periodontal disease, with reduction of LTB4, TNF and IL-12 levels, attenuation of inflammation, reduced osteoclasts number and consequently, decreased bone resorption^48,49^.

It is known that the metabolism of 5LO occurs predominantly in the nuclear membrane, and that depending on its location in the cell, cytosol, nuclear membrane, or nucleus, it indicates activation or inactivation cell status^50^. It is important to highlight that cytosolic and nuclear membrane immunolabeling were predominant in active osteoblasts. As osteoblasts differentiated into osteocytes and became entrapped in bone matrix, mild cytosolic expression was observed (Fig. 5). Yet, most of nBPs are known to improve osteoblasts and osteocytes proliferation and function, it was demonstrated that continuous expose to ZL can lead osteoblasts to caspase-associated apoptosis in humans^51^. ZL may have probably slightly impaired osteoblasts differentiation and collagenous matrix deposition in the initial periods of bone repair. Our findings for Runx2 immunolabeling demonstrated a significant decrease in Runx2 + cells at 7 days in all ZL treated groups (Fig. 7). At 21 days, WT-ZL presented a decreased expression of Runx2 compared to controls, but not 5LOKO ZL mice. In fact, ZL effect on osteoblasts seemed to be surpassed by 5LOKO animals, and satisfactory results were obtained in the late period of analysis. It is also reasonable to hypothesize that the increased expression of COX2 in 5LOKO animals (controls and ZL) benefits the osteogenic response in these groups.

Taken together, the results generated by this study demonstrated that genetic deletion of 5LO in 129 Sv female mice leads to a beneficial bone phenotyping as the animal age, as these effects are comparable to the changes induced by cumulative administration of ZL in aged WT females. Interestingly, 5LO deletion improved the capacity for dental socket healing and attenuated the ONJ under ZL administration in the aged females 129 Sv mice. We also observed that an *osteopetrotic*-like skeletal phenotype was not a risk factor for the development of ONJ related or not to the use of the nBP ZL. Finally, our findings elucidated how far ONJ pathogenesis is directly involved with osteoclasts suppression in aged individuals, as well how the genetic deletion of 5-LO impacts this pathological condition. Future mechanistic studies are necessary in order to better investigate both COX-2 and 5LO pathways in ONJ development related to ZL administration, considering osteoblasts differentiation and macrophages polarization.

## Conclusions

Our results indicated that inhibition of 5LO seems to positively influence intramembranous alveolar bone repair of aged female mice, with a direct impact on inflammatory response and bone metabolism. Considering ONJ-like lesions development related to ZL, distinct ONJ patterns could be observed in WT and 5LOKO aged female mice, resulting in a ONJ-like condition in WT animals and more attenuated ONJ-like lesions in 5LOKO mice. Taken together, these data also reinforce that ONJ onset and development related to the use of nBP ZL is highly associated with disruption of inflammatory response and bone cells, not only osteoclast suppression and osteopetrotic phenotype. Future studies are necessary to explore the mechanisms associated with the attenuation of ONJ-like lesions in this animal model.

## Materials and methods

### Animals and study design

We utilized forty female 129/SvEv wild type (WT) and 5LO (knockout for 5LO^tm1Fun^) designated here as 5LOKO aged mice, at starting age of 64 weeks and average weight of 25 g. Animals (4 mice per cage) were housed at the Animal Facility of Universidade Sagrado Coração (Bauru, SP, Brazil), at controlled temperature (22 ± 2ºC) and light cycle (12 h dark/light), with no restriction of water and food intake. All animal procedures were performed under the approval of IACUC protocol # 9589271017, following ARRIVE (Animal Research: Reporting of In Vivo Experiments) guidelines and in according to the Guide for the Care and Use of Laboratory Animals of the National Institutes of Health (Institute of Laboratory Animal Resources (U.S.)^52^. Before initiating the experiment, estrous cycle of each animal was analyzed has recommended by literature^25,53^). Next, animals were distributed into four groups, according to each genotype and treatment: WT Control, WT ZL, 5LOKO Control, and 5LOKO ZL. Mice treated with ZL received 250 μg/Kg of this drug (Merck KGaA, SML0223, Darmstadt, Germany), intraperitoneal (IP), diluted in 0.1 mL of sterile 0.9% saline solution (SS). Those belonging to control groups received only 0.1 mL of SS IP. Both solutions were administered once a week during four consecutive weeks before tooth extractions, and continuing until the end of the experimental periods, set at 7 and 21 days after the surgeries, when animals were euthanasia for sample collection. Supplementary Table 1 summarize number of animals used for each type of microscopic assay. After sample collection, groups were named with codes to minimize bias during analyses.

### Surgeries for tooth extraction

Surgeries were performed by a single and blinded surgeon, following ARRIVE guidelines. Procedures for animal care and surgical technique was performed as previously described^25^. In brief, animals received general anesthesia by intramuscular injection of ketamine chloride (80 mg/kg) (Dopalen, Agribrans do Brasil LTDA, SP, Brazil) and xylazine chloride (15 mg/kg) (Anasedan, Agribrands do Brasil LTDA, SP, Brazil). Subsequently, right upper incisor extraction was performed by carefully displacing the tooth with using a dental exploratory probe number 5 and removing the incisor with a micro forceps (Fine Surgical Instruments for Research™, Canada). As standardized in our previous studies, teeth that were fractured during extraction and also those presenting apical stem cell niche were excluded from sampling^25^. After surgery, animals received soft diet for 3 days, to avoid discomfort and pain during mastication. Animals were monitored for pain, infection and distress until the end of experimental period. At the end of experimental time points (7 and 21 days), mice of all groups were euthanized for collection of maxillae containing the post tooth extraction sockets. Additionally, 5L vertebrae and femur were collected to evaluate the skeletal phenotype of WT and 5LOKO aged females, as well the systemic effects of ZL treatment on these bones. Specimens were immediately fixed in PBS-buffered formalin (10%) solution (pH 7.4) for 48 h at room temperature, subsequently washed overnight in running water and maintained temporarily in 70% hydrous ethanol for microCT scanning. Maxillae were then decalcified in 4.13% EDTA (pH 7.2) for histological processing.

### Micro CT

Maxillae, femurs and L5 vertebrae were scanned for morphological analyses as previously described^25,29^. Specimens were rehydrated in saline solution for 10 min before scanning. Samples were scanned with Skyscan 1174 System (Skyscan, Kontich, Belgium) at 50 kV, 800 μA, with a 0.5 mm aluminum filter, 180 degrees of rotation and exposure range of 1 degree and a resolution of 14 μm pixel size. Scans were reconstructed using NRecon software (Skyscan, Kontich, Belgium), realigned in Dataviewer (Skyscan, Kontich, Belgium) and three-dimensional images were obtained by CTVox (Skyscan, Kontich, Belgium). Quantitative variables were assessed using CTAn software (Skyscan, Kontich, Belgium) to examine of socket bone healing and also for skeletal phenotyping of femur and L5 vertebrae^25,29,54^. Evaluated variables comprised fraction of Bone Volume /Tissue Volume (BV/TV, %), trabecular number (Tb.N), trabecular thickness (Tb.Th), and trabecular separation (Tb.Sp). The cortical area of the femur was evaluated considering cortical bone area (Ct.Ar, mm2).

### Histological analysis

After MicroCT scanning, maxillae containing the alveolar sockets were then decalcified in buffered 4.13% EDTA (pH 7,2) during approximately 3 weeks. Samples were embedded in paraffin blocks and sectioned in the transversal direction, as explained in Supplementary Fig. 1. At least 5 histological slices (technical replicates) of 5 µm-thick from the central region of the alveolar sockets were stained with H&E^25^ 5 obtained for modified Goldner Trichrome+Alcian Blue^55^, Picrosirius red for birefringence, and different targets by immunohistochemistry technique^25^ (Supplementary Fig. 1). Bone specimens were analyzed by2 calibrated examiners (C.C.B and M.A.M) at 2-time intervals (2 weeks apart) for histopathological investigation. Histomorphometry analysis was performed from the middle regions of the alveolar socket considering the parameters inflammatory cells, fibers and fibroblasts, osteocytes, empty lacunae and bone matrix. Histological fields were captured at 100 × using oil immersion objective (Carl Zeiss Jena GmbH, Jena, Germany). Then, a grid image containing a total of 100 points was superimposed on each histological field by using ImageJ software (Version 1.51, National Institutes of Health, Bethesda, Maryland, USA). The intersection of the vertical and horizontal lines coincident with the histological parameters was considered a point, and the total number of points was obtained to calculate the area density for each parameter. All groups were coded for a blinded analysis by each calibrated co-author. Results were presented as mean + /- standard deviation (SDs) of the area density.

### Birefringence analysis for collagenous content

Histological sections stained with Picrosirius red were visualized and captured with a 10 × objective by using a polarizing lens coupled to a binocular inverted microscope (Leica DM IRB/E), as previously described^22^. Control pictures were captured using conventional light. Spectra of green, yellow and red colors were defined by RGB values and the quantity of pixels^2^ was calculated for each field by using AxioVision 4.8 software (Carl Zeiss). After calculation of the area of green (thin and immature fibers), as well as yellow and red collagen fibers (thicker and mature), the total area was also calculated by the sum of each color spectrum. Mean + /- standard deviation (SD) considering the 2 technical replicates (histological fields) and 5 biological replicates (*n* of animals *per* group) were calculated for each genotype, time point and experimental group. All groups were coded for a blinded analysis by each calibrated co-author.

### Picro-thionin (Schmorl) staining for the analysis of osteocytes lacunae

For the analysis of osteocytes’ lacunae, femurs were stained with picro-thionin dye. Histological slices were re-hydrated, washed in tap water and immersed in 0.125% thionin for 15 min, and then washed again and immersed in 1.3% picric acid. Afterwards, the slices were dehydrated in alcohol followed by two bathes with xylol for 3 min each. The images were captured in a 40 × magnification, and four fields of the cortical bone between periostal and endostal surfaces of distal metaphysis were chosen. Each lacuna was manually quantified using histomorphometric analysis as described for H.E staining. All groups were coded for a blinded analysis by each calibrated co-author.

### Immunohistochemistry and histomorphometric analysis for inflammatory factors and bone healing markers

After histological analysis of all groups, all samples were used for immunohistochemistry to analyze TRAP (sc#30832), Runx2 (sc#8566) and F4/80 + cells (sc# 26643), OCN (#sc-18319), COX 2 (#sc-1747), and 5LO (sc -136195). In general, histological sections of the alveolar socket were deparaffinized following standard procedures. Slices were pre-incubated with 3% Hydrogen Peroxidase Block (Spring Bioscience Corporation, CA, USA) and subsequently incubated with 7% NFDM to block serum proteins, as previously described^25^. Primary antibodies were purchased from Santa Cruz (Santacruz Biotechnology, Carpinteria, USA), and samples were incubated at a concentration of 1:100 for 1 h at room temperature. For detection methods, a universal immuno-enzyme polymer method was used and sections were incubated in immunohistochemistry staining reagent for 30 min at room temperature. For detection of antigen–antibody was used 3–3'-diaminobenzidine (DAB), followed by counter-staining with Mayer's hematoxylin. For 5LO staining, counterstaining was performed using Fast green for 30 min. After completing the immunolabelling, six histological fields per histological section were captured using a 100 × oil immersion objective (Carl Zeiss Jena GmbH, Jena, Germany). Positive cells for each marker were counted by a single calibrated investigator as previously described for H&E staining. All groups were coded for a blinded analysis by each calibrated co-author.

### Statistical analysis

Quantitative data were first analyzed for distribution of normality using Shapiro–Wilk normality test. The effect of genotype and/or drug treatment for quantitative parameters were analyzed by One-way ANOVA with post-hoc Tukey test for multiple comparison. For data with non-normal distribution, Mann–Whitney and/or Kruskal–Wallis (followed by the Dunn's test) tests were used for comparison between 2 groups or multiple comparison respectively, when applicable. The alpha level for all tests was set to (5%), then p < 0.05 was considered statistically significant. Data sets were presented with descriptive statistics, containing mean and standard deviation (SD) or Box and whiskers (Min to Max). All statistical tests were performed using the GraphPad Prism 5.0 software (GraphPad Software Inc., San Diego, CA, USA).

## Supplementary Information

Supplementary Information 1.

Supplementary Figure 1.

Supplementary Figure 2.

Supplementary Information 2.
