# Supplementary material for: Alveolar socket healing in 5-lipoxygenase knockout aged female mice treated or not with high dose of zoledronic acid
Source: Sci Rep. 2021 Oct 1;11:19535. doi: 10.1038/s41598-021-98713-2 (PMC8486749; doi:10.1038/s41598-021-98713-2)
Supplement: Supplementary file 1 — Supplementary Information 1. [file 41598_2021_98713_MOESM1_ESM.doc]

| **Type of analysis** | **Outcome** | **Sites** | **Intervention** | **Genotype** | **Healing period** | **Result** |
| --- | --- | --- | --- | --- | --- | --- |
| **microCT and**  **Histology (HE and Picro-thionin)** | **Skeletal phenotype** | L5, femur metaphysis and diaphysis | ZL treatment for 8 weeks | WT and 5LOKO | N/A | Table 1, Figure 1 |
| **microCT** | **Newly formed** | Maxillae | Tooth extraction and ZL treatment for 5 (7d) and 8 weeks (21d) | WT and 5LOKO | 7 and 21 days | Figure 2 |
|  | **mineralized bone** |  |  |  |  |  |
| **Histology and Histomorphometry** | **Bone healing** | Maxillae | Tooth extraction and ZL treatment for 5 (7d) and 8 weeks (21d) | WT and 5LOKO | 7 and 21 days | Figure 3 and Table 2 |
| **(HE and TG)** | **or MRONJ**  **development** |  |  |  |  |  |
| **Birrefringence**  **analysis** | **Quality of Bone**  **Matrix** | Maxillae | Tooth extraction and ZL treatment for 5 (7d) and 8 weeks (21d) | WT and 5LOKO | 7 and 21 days | Figure 4 |
|  |  |  |  |  |  |  |
| **IHQ** | **5LO** | Maxillae | Tooth extraction and ZL treatment for 5 (7d) and 8 weeks (21d) | WT | 21 days | Figure 5 |
|  | **F4/80, Cox2**  **(Inflammation markers)** |  |  | WT and  5LOKO | 7 and 21 days | Figure 6 |
|  | **TRAP, Runx2 and OCN (Bone markers)** |  |  |  | 7 and 21 days | Figure 7 |
