# Supplementary figures and images for: Alveolar socket healing in 5-lipoxygenase knockout aged female mice treated or not with high dose of zoledronic acid
Source: Sci Rep. 2021 Oct 1;11:19535. doi: 10.1038/s41598-021-98713-2 (PMC8486749; doi:10.1038/s41598-021-98713-2)

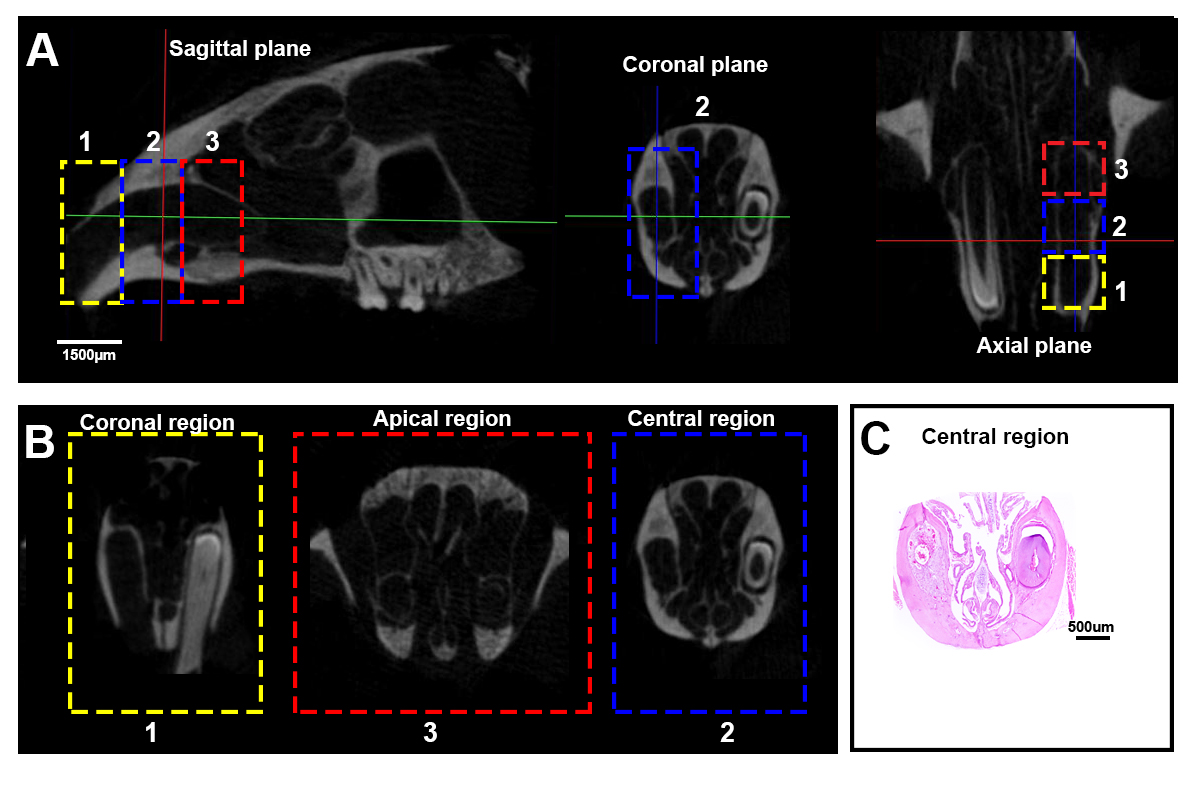

Supplement: Supplementary file 2 — Supplementary Figure 1. [file 41598_2021_98713_MOESM2_ESM.jpg]

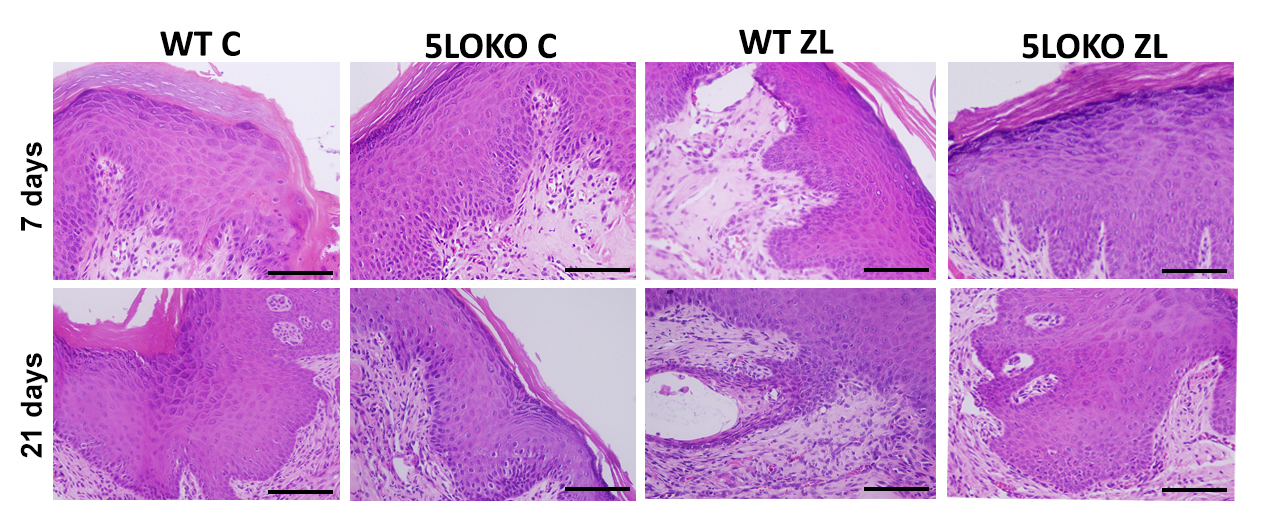

Supplement: Supplementary file 3 — Supplementary Figure 2. [file 41598_2021_98713_MOESM3_ESM.jpg]
