# Supplementary material for: Alveolar socket healing in 5-lipoxygenase knockout aged female mice treated or not with high dose of zoledronic acid
Source: Sci Rep. 2021 Oct 1;11:19535. doi: 10.1038/s41598-021-98713-2 (PMC8486749; doi:10.1038/s41598-021-98713-2)
Supplement: Supplementary file 4 — Supplementary Information 2. [file 41598_2021_98713_MOESM4_ESM.docx]

**Legends of Supplementary Figures**

**Supplementary Figure 1.** Representation of maxilla from an aged 129Sv female mice with regions of interest used for histomorphometric analysis. A) Microtomographic reconstructions in 2D view demonstrated the 3 regions of alveolar socket: 1 – coronal region, 2 – central region, 3- apical region. Each region is demonstrated in sagittal, coronal and axial planes. B) The three regions of alveolar socket are demonstrated in coronal plane, which is the same used for transversal sections in H&E. C) H&E stained slice showing a transversal/coronal section of 129SV aged female maxilla.

**Supplementary Figure 2.** Representation of epithelialization on alveolar socket healing in WT and 5LOKO mice. H&E staining. Scale bar = 100µm
